# Association between MGMT status and response to alkylating agents in patients with neuroendocrine neoplasms: a systematic review and meta-analysis

**DOI:** 10.1042/BSR20194127

**Published:** 2020-03-25

**Authors:** Zhirong Qi, Huangying Tan

**Affiliations:** Department of Integrative Oncology, China-Japan Friendship Hospital, Beijing 100029, People’s Republic of China

**Keywords:** alkylating agents, meta-analysis, neuroendocrine neoplasms, O6-methylguanine-DNA methyltransferase

## Abstract

**Background:** O^6^-methylguanine-DNA methyltransferase (MGMT) is a specific DNA damage reversal repair protein. The influence of MGMT status on alkylating agent sensitivity in patients with neuroendocrine neoplasms (NENs) is controversial. We conducted a meta-analysis to assess the influence of MGMT status on the therapeutic sensitivity of alkylating agents in patients with NENs.

**Methods:** We searched PubMed, EmBase, and Cochrane library public databases through 3 July 2019. The objective response rate (ORR) was the outcome data of interest. Subgroup analysis was performed according based on MGMT methylation and expression of MGMT protein.

**Results:** Eleven studies were included in the meta-analysis. The proportion of patients with NENs that achieved an ORR after alkylating agent treatment was higher in the MGMT-deficient group than the non-deficient group (OR: 5.00; 95% CI: 3.04–8.22; *P* < 0.001; *I*^2^: 3%). Similar results were noted in the MGMT methylation and MGMT protein expression subgroups.

**Conclusion:** Patients with NENs and MGMT methylation or low protein expression had a higher ORR proportion than patients without MGMT methylation or high protein expression. The MGMT status can be used as a biological indicator of the response to alkylating agent treatment in patients with NENs.

## Introduction

Neuroendocrine neoplasms (NENs) are rarely tumors that originate from embryonic neuroendocrine cells that have neuroendocrine markers and can produce polypeptide hormones. The types of NENs are named based on site of origin and histology. They frequently detected in the gastroenteropancreatic tract and bronchopulmonary tree, and typed as pancreatic, gastrointestinal and pulmonary NENs [1]. Histologically they can be of two types: well differentiated and poorly differentiated. In Europe and the United States, the incidence of pancreatic NENs is 2.5–5 per 100,000 population [2]. In recent years, however, the incidence of NENs has increased significantly [3], possibly because the identification of molecular markers and advances in endoscopic techniques have increased the detection rate and diagnostic accuracy. Chromogranin A has been shown to be diagnostic molecular marker with good accuracy [4], but pathologic examination remains the gold standard for final diagnosis. Because of the lack of specificity of clinical symptoms, most patients have distant metastases at the time a definite diagnosis is established, and the prognosis is poor [5]. The response rate of temozolomide-based chemotherapy for advanced or metastatic NENs is 30–70% [6]. Therefore, how to judge the chemosensitivity of patients with NENs is a topic of concern.

O^6^-methylguanine-DNA methyltransferase (MGMT) is a widely studied specific DNA damage reversal repair protein that protects chromosomes from mutation, carcinogenesis, and cytotoxic effects of alkylating agents [7]. In previous studies, MGMT status was shown to be related to the occurrence and invasion of non-small cell lung cancer (NSCLC) [8], melanoma [9], malignant glioma [10], esophageal cancer [11], and prostate cancer [12]. MGMT also affected the chemotherapeutic effect of different tumor patients, but the results are controversial [8,9,13].

Alkylating agents, such as temozolomide, dacarbazine, and streptozotocin, can form DNA cross-linking to block DNA replication, thus killing tumor cells, and are often used as second-line therapy for NENs [14]. MGMT can inhibit the killing effect of alkylating agents on tumor cells by chromosome protection [15]. Thus, the status of MGMT expression may be related to tumor chemosensitivity; however, the relationship between the sensitivity of NENs to alkylating agents and MGMT status is controversial. For example, Kulke et al. researched 21 tumor samples from NENs patients who received temozolomide treatment. It found 4 of 5 MGMT-deficient patients get tumor response after treatment, and none of 16 MGMT-proficient patients get tumor response. So, they considered there is a correlation with MGMT expression and treatment response [16]. However, Raj et al. researched effect of Alkylating agents on pancreas treatment. It found 10 of 20 MGMT-deficient patients get partial response, 5 of 16 MGMT-intact patients get partial response. And then it also found 1 of 4 MGMT promoter methylation positive patients get partial response, and 9 of 24 negative patients get partial response. So they concluded MGMT status should not guide alkylating agent treatment for well-differentiated pancreatic NENs patients [17]. In this meta-analysis, we assessed the influence of MGMT expression on the therapeutic sensitivity of patients with NENs to alkylating agents.

## Methods

### Data sources and search strategy

We used the keywords, “MGMT,” “methylguanine-DNA methyltransferase,” “neuroendocrine,” “carcinoid,” “methylation,” “hypermethylation,” “methylated,” and “epigene,” to identify related articles in PubMed, EmBase, and the Cochrane library. The retrieval deadline was 3 July 2019. The references of related reviews were also searched to prevent omissions.

### Selection criteria

A study was included if the following criteria were met: 1. case–control study design; 2. involved patients with NENs; 3. compared patients with positive and negative MGMT promoter methylation or MGMT protein expression; and 4, reported the ORR outcome with original data of each group. The exclusion criteria were as follows: 1. included other type patients; 2. MGMT gene methylation or protein expression status was not studied; and 3. no ORR or raw data were reported.

### Data extraction

Two authors extracted the information from the included studies and any inconsistencies were resolved through discussion. The following information was extracted from each study: first author’s name; year of publication; country; patients’ average age; stage of disease; and MGMT testing methods. The main outcome was the ORR, which included complete plus partial responses. The Newcastle–Ottawa Scale (NOS), which includes three subscales (selection, comparability, and exposure), was used to evaluate the methodologic quality of the included studies [18].

### Statistical analysis

Odds ratios (ORs) and 95% confidence intervals (CIs) were used to calculate the strength of the association between MGMT status and ORR. The *I*^2^ statistic was taken as measures of heterogeneity among the included studies. If the *I*^2^ was ≤ 50%, the fixed-effect model was used; otherwise, the random-effect model was applied. Subgroup analysis was also performed according to the detection of MGMT methylation and MGMT protein expression. Funnel plots were used to evaluate publication bias. All tests were two-tailed, and a *P* value < 0.05 was considered statistically significant.

## Results

**Figure 1:**
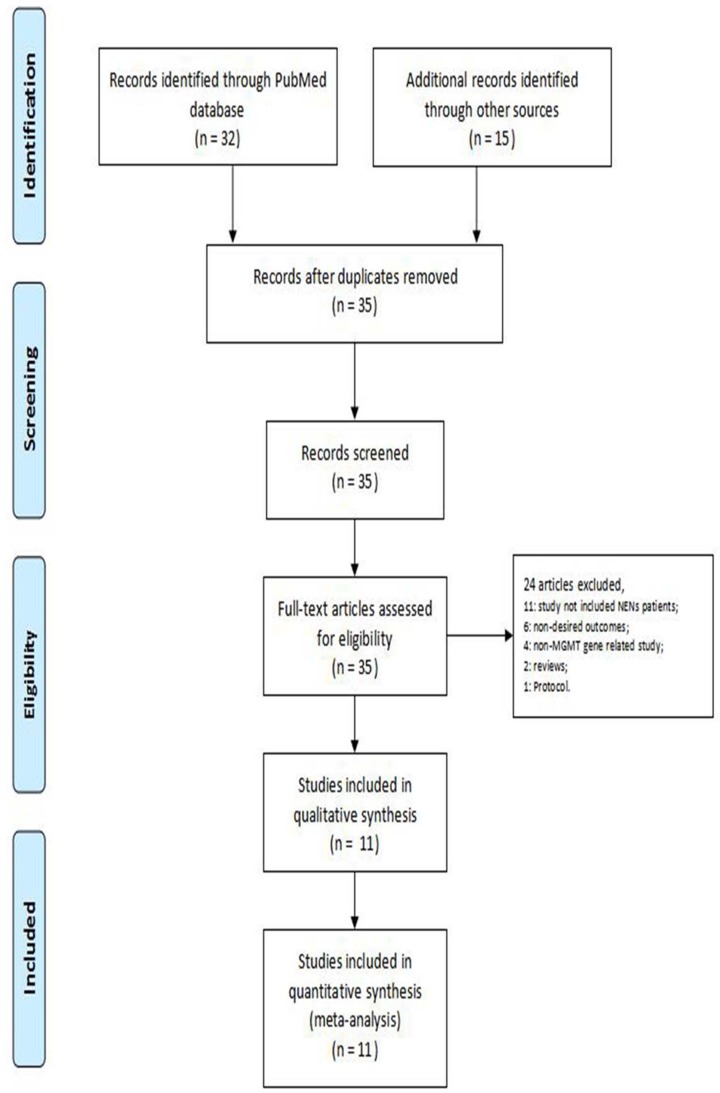
PRISMA flow chart illustrating the selection of studies included in our analysis

The literature search yielded 35 records. Thirty-five full-text articles were further assessed. The following articles were excluded for the following reasons: NENs patients were not included (*n* = 11); undesired outcomes (*n* = 6); non-MGMT gene study (*n* = 4); reviews (*n* = 2); and study protocol (*n* = 1). Therefore, 11 studies were included in this meta-analysis [16,17,19–27] (Figure 1 and Table 1).

**Table 1:** The characteristic of included studies

Author (Publication year)	Country	Primary location	Therapy	Mean or median^#^	Stage of disease	Method	NOS score
Walter et al.(2015) [16]	France	Pancreas 58%; GI tract 31%; Lung 4.6%; Other 6.5%.	Alkylating Agents	53	Advanced well-differentiated NETs	PSQ	7
Campana et al.(2017) [17]	Italy	Pancreas 45%; Lung 23%; Other 32%.	Temozolomide	62	Metastatic NENs	PSQ/MSP	8
Welin et ai.(2011) [18]	Sweden,Norway	Pancreas 40%; Colorectal 20%; Gastric 8%; Bronchial 12%; Unknown 20%.	Temozolomide	55	Metastatic PDEC	MSP	6
Raj et al.(2017) [19]	U.S.A.	Pancreas 100%	Alkylants	55	Locally advanced or metastatic NETs	MSP/IHC	7
Owen et al.(2017) [20]	U.S.A.	Pancreas 61%; Lung 8%; Rectum 8%; Stomach 3%; Adrenal 3%; Tonsil 3%; Ovary 3%; Thyroid 3%; Carotid Body 3%; Unknown 8%.	Temozolomide	53	Metastatic NETs	IHC	6
Dussol et al.(2015) [21]	France	Pancreas 43%; GI tract 33%; Lung 14%; Other/unknown 10%.	Alkylating Agents	60	Advanced well-differentiated NETs	MSP/IHC	7
Kulke et al.(2009) [22]	U.S.A.	Pancreas 100%	Temozolomide	57	Locally advanced or metastatic NETs	IHC	7
KRUG et al.(2017) [23]	Germany	Pancreas 83.3%; Bronchus 8.3%; Gastric 4.2%; Midgut 4.2%.	Alkylating Agents	53	Advanced NETs	IHC	8
Cros et al.(2016) [24]	France	Pancreas 100%	Temozolomide	58	Locally advanced or metastatic NETs	IHC	6
Cives et al.(2017) [25]	U.S.A.	Pancreas 100%	Temozolomide	59	Locally advanced or metastatic NETs	IHC	7
Hijioka et al.(2018) [26]	Japan	Pancreas 100%	Streptozocin	64	Advanced NETs	IHC	7

Abbreviations: GI, gastrointestinal; IHC, immunohistochemistry; MSP, methylation-specific polymerase chain reaction; NE, neuroendocrine tumours; NEN, neuroendocrine neoplasm; NOS, Newcastle–Ottawa Scale; PDEC, poorly differentiated endocrine carcinoma; PSQ, pyrosequencing.

# Units: Year

The included studies were published between 2009 and 2018. The studies mainly included patients with locally advanced or metastatic NENs. The patients received alkylating agent treatment, including streptozotocin, temozolomide, and dacarbazine. Immunohistochemistry at the protein level, pyrosequencing (PSQ) analysis, and methylation-specific PCR (MSP) detection were used for MGMT status detection. Two studies reported protein level and epigenetics results at the same time [19,21]. Evaluation of study quality showed that the highest and lowest scores were 8 and 6, respectively. The main factor affecting quality was a lack of comparison of the characteristics among the different MGMT status groups. Another factor was a lack of unified criteria for intergroup classification based on IHC protein expression; however, the overall design quality of the included studies was ideal (Table 1).

**Figure 2:**
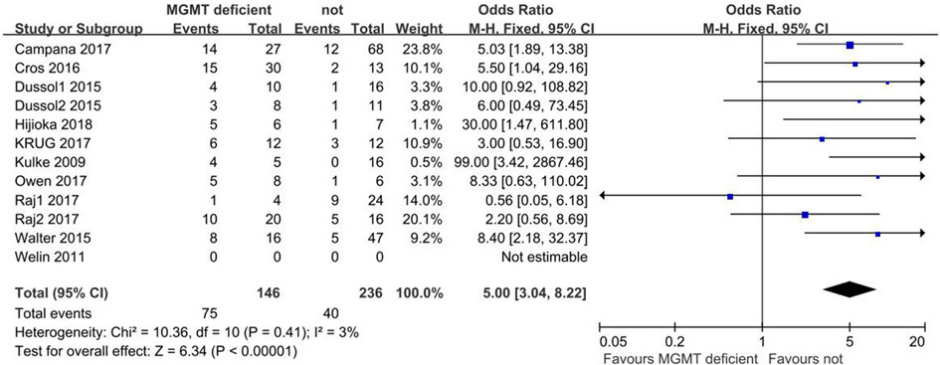
Forest plot of ORR results comparing MGMT deficient and non-MGMT deficient patients with NENs after alkylating agent treatment

**Figure 3:**
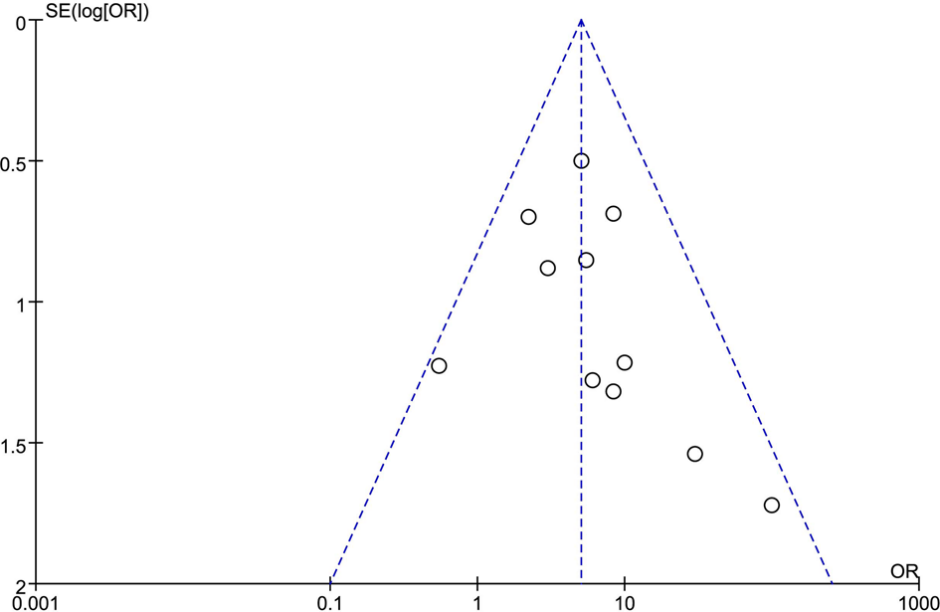
The funnel plot for assessing potential publication

MGMT deficiency rather than the population was compared to generate ORR results. Due to the low heterogeneity among comparisons, the fixed-effect model was used. Pooled results showed the proportion of patients with NENs who achieved ORR after alkylating agent treatment was higher in the MGMT-deficient group than the non-deficient group (OR: 5.00; 95% CI: 3.04, 8.22; *P* < 0.001; *I*^2^: 3%; Figure 2). Funnel plots showed that no significant publication bias exists (Figure 3).

**Figure 4:**
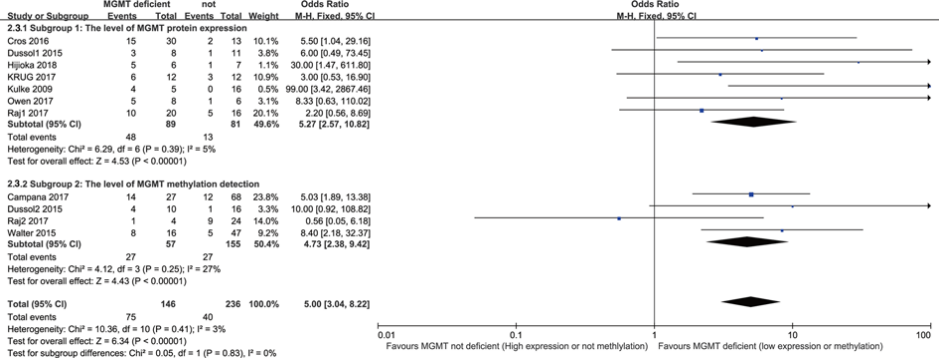
Subgroup analysis of ORR results according to MGMT protein expression (subgroup 1) and detection of MGMT methylation (subgroup 2)

According to different levels of MGMT status detection, we separated the results of methylation and protein levels based on subgroup analysis. The level of protein expression results showed that patients with NENs and low expression of MGMT protein had a higher proportion of ORR compared with high expression of MGMT protein after alkylating agent treatment (OR: 5.27; 95% CI: 2.57, 10.82; *P* < 0.001; *I*^2^: 5%). The level of MGMT methylation detection showed that the proportion of patients with NENs and MGMT promoter methylation who reached ORR was greater than the proportion of patients without methylation after alkylating agent treatment (OR: 4.73; 95% CI: 2.38, 9.42; *P* < 0.001; *I*^2^ = 3%). There were similar results between the subgroups (Figure 4).

## Discussion

This meta-analysis analyzed the relationship between MGMT deficiency status and ORR results in patients with NENs who received alkylating agent treatment. The results showed that the patients with NENs and MGMT methylation or low protein expression had a higher ORR than patients without MGMT methylation or high protein expression. The MGMT status can be used as a biological indicator of the response to alkylating agent treatment in patients with NENs.

NENs is a relatively rare kind of tumors. In Europe and the United States, the incidence of pancreatic NENs is only 2.5–5 per 100,000 population [2]. Therefore, compared with other types of tumors, neuroendocrine tumors are still relatively less studied. Therefore, the lack of NENs and MGMT status related research led to the less number of literature records in this meta-analysis.

The main biological function of MGMT is a DNA repair enzyme that prevents the formation of DNA cross-linking and reduces the cytotoxic effect of alkylating agents. MGMT status might affect the treatment effect from the mechanism [7]; our work only researched the influence of alkylating agents in patients with NENs based on MGMT status. Whether or not-other types of drug treatment responses, such as everolimus related regimens, are significantly affected by MGMT status warrants further study [28]. MGMT may play a very important role in carcinogenesis and invasive risk. It has been shown that MGMT hypermethylation is associated with an increased risk of NSCLC [8]. MGMT promoter methylation is also associated with the occurrence and invasion of melanoma [9]. Furthermore, MGMT methylation can also be used as a biomarker to predict carcinogenesis in a variety of tumors [29–32].

The drug resistance of NENs cells to alkylating agents can be regulated by MGMT. In general, MGMT promoter methylation or epigenetic silencing can reduce DNA repair capacity, and therefore improve chemotherapy sensitivity that benefits survival, but also results in an increased risk of new carcinogenesis. Patients without MGMT methylation had a low risk of carcinogenesis because of high MGMT expression and an advanced DNA repair effect; however, when a tumor is established, the effect of chemotherapy and life expectancy are poor. Thus, patients with MGMT methylation are prone to tumors and tumor invasion, but the treatment effect is good. Demethylated patients are less likely to develop tumors, but when tumors are established, the sensitivity to chemotherapy is poor. Therefore, MGMT is more suitable to predict survival in patients and the choice of chemotherapy drugs. Because of the potential impact on normal cells, MGMT may not be suitable as a potential drug targeting site.

Although targeted drugs are currently used in NENs, the alkylating agent alone or in combination with other type’s drugs is still an important way of NENs treatment [19]. Such as the temozolomide used alone or in combination with other drugs obtain a high response rate in NENs treatment with high safety [21]. For the lung NENs, temozolomide as a palliative treatment drug is also widely used and has an acceptable safety profile [33]. Generally, 25–50% of NENs are accompanied by promoter methylation of MGMT. According to the different primary sites, it is estimated that about 50% MGMT methylation in pancreatic NENs, while MGMT methylation is approximately 0–15% in lung and gastrointestinal NENs [16,19,25]. Different MGMT status of primary sites may be the reason for the different sensitivity of NENs to alkylating agent treatment. To improve effect of NENs treatment in clinic, one way is to find novel effective agents, and the other is to select relative effective drugs according to patient characteristics, such as MGMT status. Therefore, it is very important to clear the association between response rate of alkylating agents in NENs patients and MGMT status. The present study confirmed the viewpoint that the MGMT status can be used as a biological indicator of the response to alkylating agent treatment in patients with NENs.

### Limitation

There were several limitations in this work. First, the present study was performed based on the study level, not the individual level. Second, the lack of analysis in the follow-up period may have affected the pooling results. Third, the level of MGMT protein expression was high and low level among the included studies, but there is still no uniform standard for group division. Fourth, in addition to MGMT status, the treatment effect may also be affected by the location of the primary tumor, the extent of metastasis, and the response to surgery. The above factors will also affect the final results.

## Abbreviations

CI: confidence interval
MGMT: O^6^-methylguanine-DNA methyltransferase
NEN: neuroendocrine neoplasm
OR: odds ratio
ORR: objective response rate
